# The Application of ASSRs, P50, and MMN in the Exploration of Cognitive Dysfunction Involving Inputs and Processing in Insomnia Patients

**DOI:** 10.3389/fnhum.2021.714302

**Published:** 2021-09-03

**Authors:** Hongwei Zhao, Chang Liu, Changming Wang, Xiangyu Zheng, Yanhui Peng, Yudan Lv

**Affiliations:** ^1^Department of Neurology and Neuroscience Center, The First Hospital of Jilin University, Changchun, China; ^2^Department of Neurosurgery, Xuanwu Hospital, Capital Medical University, Beijing, China; ^3^Department of Neurology, The Sixth Affiliated Hospital of Xinjiang Medical University, Urumqi, China

**Keywords:** insomnia, cognitive dysfunction, ASSRs, P50, MMN

## Abstract

Objective cognitive dysfunction has been commonly found in patients with insomnia, such as attention, memory, speed of information processing, and executive functions. Auditory steady-state responses (ASSRs), P50, mismatch negativity (MMN) can meet varied need and estimate such different cognitive dysfunction. Thus, we can examine whether insomnia is associated with different cognitive dysfunction by such multiple event-related potential (ERP) tasks. Methods we used polysomnography (PSG) to record such objective PSG parameters. ASSR, P50, and MMN were performed in sequence, different ERP components have been analyzed such as latency or amplitude between insomnia group and control group. And we chosed person correlation to make correlation analysis between different ERP components and gender, education, and sleep characteristics. Results there is a significant gender difference of ASSR latency found in insomnia group, and the similar result has been found in suppression ratio of amplitudes (S2:S1) for P50. Additionally, a significant correlation between sleep characteristics and ASSR, P50 has been found. Furthermore, there was a significant difference of MMN latency between insomnia and control group, and between sleep characteristics and varied MMN parameters as latency and amplitude. Discussion our results suggested robust electrophysiological abnormalities as ASSR, P50, and MMN in insomnia patients. Such abnormalities included gender difference, education difference, difference in depressive tendency, and difference in sleep parameters. That results revealed varied cognitive dysfunction involving inputs and processing in insomnia patients. And at the same time, we have also explored the neuropsychological mechanisms underlying the cognitive dysfunction with such different ERP tasks.

## Introduction

Insomnia is a kind of sleep disorder syndrome, in which the physiological needs of individuals are not met due to insufficient sleep time or poor sleep quality, which in turn significantly affects activities during the day (Coolidge and Segal, 1998). Epidemiological studies showed that the prevalence of insomnia in the global population is 30–48% (Ohayon, 2002). In China, 45.4% of people have experienced insomnia, and 10% suffer from insomnia (Soldatos et al., 2005). It is a common complication and a predisposing factor of mental disorders, cardiovascular or cerebrovascular diseases, and other diseases.

Furthermore, cognitive dysfunction has been commonly found in patients with insomnia, such as attention, memory, speed of information processing, and executive functions (Fulda and Schulz, 2001). The use of event-related potentials (ERPs) would provide a more direct measure of sensory and cognitive processing in insomnia, which can provide a task-time related analysis and even reveal the correlations between psychophysiology and poor performance.

Among ERP studies, the so-called auditory steady-state responses (ASSRs), P50, and mismatch negativity (MMN) are of particular interest and can estimate different cognitive dysfunction involving inputs, integration, processing, execution, and so on. ASSRs (Ross et al., 2005), generated in the primary auditory cortex, have been widely used to assess neural synchrony and the integrity of auditory pathways within and between cortical regions, which represented information integration function. Sensory gating P50 (Freedman et al., 1991) refers to the ability of cerebral networks to transmit incoming information and filter out irrelevant stimuli, which represented the information filtering function and can protect the brain from information overflow. MMN reflects a neurophysiological mismatch between perceptual inputs and preceding sensory information stored in the short-term memory trace, which represented information matching and processing function (Naatanen et al., 2007).

Overall, the aim of our study was to examine whether insomnia is associated with different cognitive dysfunctions (detected by ASSRs, P50, and MMN) and whether different ERPs would detect similar cognitive dysfunction. Under this situation, the following questions are intended to be answered: Firstly, whether different parameters in ERP measures as amplitudes or frequencies were distinguished differently between patients with insomnia and controls; Secondly, whether ERP measures are correlated with clinical variables such as psychotic symptoms, insomnia severity, and sleep quality; and finally, whether cognitive dysfunction in patients with insomnia is correlated with specific brain regions.

## Materials and Methods

The study was approved by the Ethics Committee of Jilin University First Hospital, and all participants signed their informed consent.

### Participants

Two groups were compared: patients with insomnia and healthy controls. There were 30 insomnia patients and 30 healthy controls, and both groups were matched in age, gender, and education.

Inclusion criteria were: (1) All patients with insomnia have had difficulty in falling asleep, are easy to wake up, and have difficulty in falling asleep again after waking up early, for at least 1 month; (2) Such patients have no other sleep disorders, such as narcolepsy, parasomnias, or other mental disorders; (3) Patients and controls are between 30 and 70 years old, with primary school education or above; (4) The patients’ score over seven on the Pittsburgh sleep quality index (PSQI), and did not take any psychotropic drugs during the study period or for 2 weeks prior; and (5) All subjects were right-handed and had no history of smoking, drug, or alcohol abuse.

Exclusion criteria were: (1) The patients’ insomnia is caused by organic diseases, or by severe depression and anxiety; (2) The patient had brain injuries such as stroke or traumatic brain injury; (3) Have severe hearing impairment; (4) Those who cannot fully cooperate with the inspection due to other factors; or (5) The insomnia patients or control subjects had AHI>5, PLMS>15.

### Polysomnography-Recordings

We used polysomnography (PSG) to record the sleep. A standard montage was used including eight EEG channels (F3, F4, C3, C4, O1, and O2), two mastoid channels (A1, A2), two electrooculogram (EOG) channels, and one submental electromyogram channel. This montage was complemented by recordings from the left and right anterior tibialis muscle, recordings of nasal/oral airflow, thoracic and abdominal effort, body position, and oximetry. All PSG were scored according to the standard criteria by an experienced sleep specialist. Objective PSG parameters have been selected as sleep efficiency (%), arousal index, total sleep time (TST, min), percentage (%) of stages 1, 2, and 3 + 4, and rapid eye movement (REM) and heart rate.

### Event-Related Potential-Recordings

During the ERP-recordings, all the participants were tested in a quiet and sound-proof room and seated in a relaxed position. Instructions were shown in the display, and technicians instructed the participants to have a Preview exercise. All electrodes were placed according to the international 10–20 System with Ag/AgCl ring. Mastoids references were used and the impedances were kept below 10 kΩ. All electrodes were referenced to Cz during recording. Eye movements were recorded by an EOG placed 1 cm below the left eye and 1 cm above the right eye.

Auditory steady-state response stimuli were presented in two blocks of stimuli (150/block) through earphones: 20- and 40-Hz stimulation rates. Stimuli consisted of trains of 1-ms white noise clicks (500-ms duration, 1,100-ms stimulus onset asynchrony, and 80 dB sound). Subjects were instructed to look at the fixation cross on the monitor and listen to the stimuli. Single-trial epochs were extracted (−250–800 ms).

P50 was collected in 1,500 ms epochs, beginning 500 ms before S1, continuing for 500 ms to S2, and terminating 500 ms after S2. Each train consisted of 600 data points with 2.5 ms resolution. P50 to the first click was selected as the most positive deflection of 40–80 ms after the click. The larger amplitude N1 was used to locate P50 latency. P50 to the second click was identified within 10 ms of its latency to the first click. Suppression ratios for P50 were calculated (stimulus2: stimulus1) on the individual data as a measure of sensory gating, which indicated suppression level between groups.

The auditory stimuli of MMN were delivered at 85 dB SPL. The subjects were asked to press a button promptly in response to target tones. The auditory stimuli in MMN consisted of 85% standard tones (50 ms, 90 dB SPL, 550 Hz sine wave, 10 ms rise, and fall times) and 15% duration-deviant tones (100 ms). The stimulus sequence was pseudorandom (deviants were preceded by at least one standard). The MMN was extracted by subtraction of the standard from the deviant ERP. The MMN component was identified as a negative wave in the 125–260 ms interval, and it was evaluated at the CZ site (where the amplitude was maximal).

### Artifact Elimination

Eye movements were recorded by an EOG placed 1 cm below the left eye and 1 cm above the right eye. A 30 Hz lowpass filter and 0.3 Hz highpass filter were applied. The averaging of the ERP waves and related procedures were performed using Curve 7.0 software (Compumedics USA, Ltd., Charlotte, NC, United States). Any bad channel or gross movement artifacts were removed from the recorded data by visual inspection, and eye blinks were removed using established mathematical procedures. Trials were rejected if they included significant physiological artifacts (amplitude exceeding ±75 μV) at all cortical electrode sites. After artifact removal, a baseline correction was conducted by subtracting the mean value for 100 ms before the stimulus onset from the post-stimulus data for each trial.

### Statistical Analysis

Statistical analysis was performed with Prism7.0 software. For PSG and ERP (ASSR, MMN, and P50) analysis, we used unpaired *t*-test. Significant interactions were conducted with Tukey’s honest significance test. The significance level was set at *p* < 0.05. For the correlation analysis, we chose Person correlation coefficient R.

## Results

### Clinical and PSG Evaluation

There is no difference in age, gender, and Body Mass Index (BMI) between the insomnia group and control group, listed in Table 1. As shown, the insomnia group has lower sleep efficiency than the control group (66.31% vs. 91.43%), higher arousal index (21.92 times/h vs. 4.6 times/h), and abnormal sleep structure with shorter N3 and REM (3.58% vs. 17.22%, 14.4% vs. 20.17%, respectively).

**TABLE 1:** Clinical characteristics and polysomnography (PSG) results obtained from insomnia group and control group.

**Demographic and clinical characteristics**	**Insomnia-group**	**Control-group**
Number of samples	30	30
Age (years)	56.75 ± 5.7	57.65 ± 6.3
Male (%)	14.29	17.41
Female (%)	85.71	82.59
Duration of insomnia (years)	7.33	–
BMI	23.16	23.75
Depression or irritability	yes	no
Sleep efficiency (%)	66.31	91.43
AHI	4.1	3.9
PLMI	8.7	7.6
Arousal index	21.92	4.6
Total sleep time (TST,min)	358.7	385
N1 (%)	27.08	16.41
N2 (%)	55.07	48.01
N3 (%)	3.58	17.22
REM (%)	14.4	20.17
Heart rate	67.53	70.65

### Evaluation Between Gender and ASSR and P50 in Insomnia Group

**FIGURE 1:**
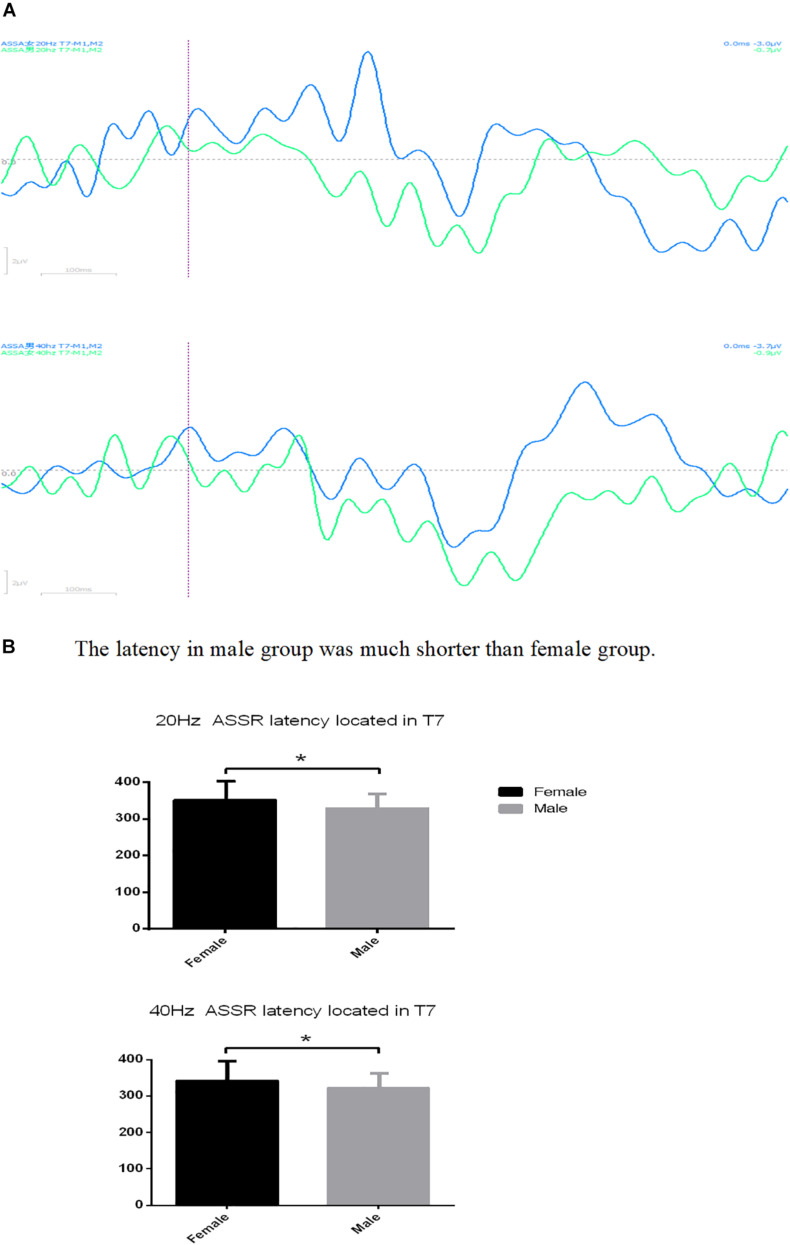
**(A)** The latency located in T7 differed between male and female in both 20 Hz auditory steady-state response (ASSR) and 40 Hz ASSR. **(B)** The latency in male group was much shorter than female group. ^∗^*P* < 0.05.

**FIGURE 2:**
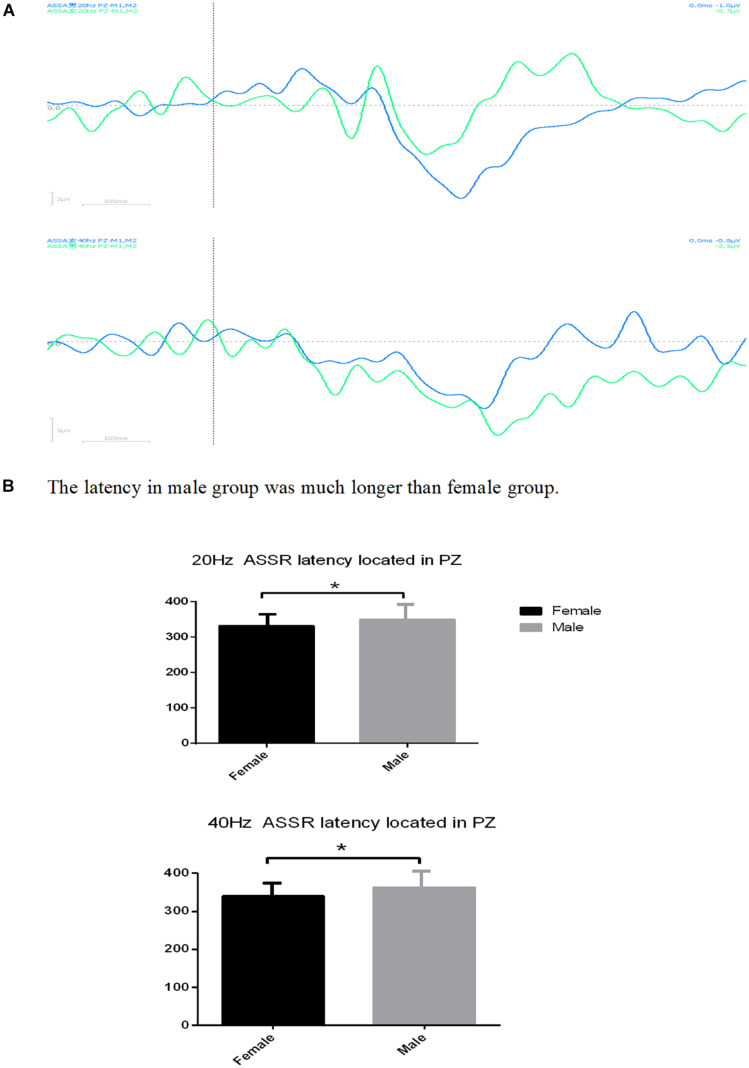
**(A)** The latency located in Pz differed between male and female in both 20 and 40 Hz ASSR. **(B)** The latency in male group was much longer than female group. ^∗^*P* < 0.05.

**FIGURE 3:**
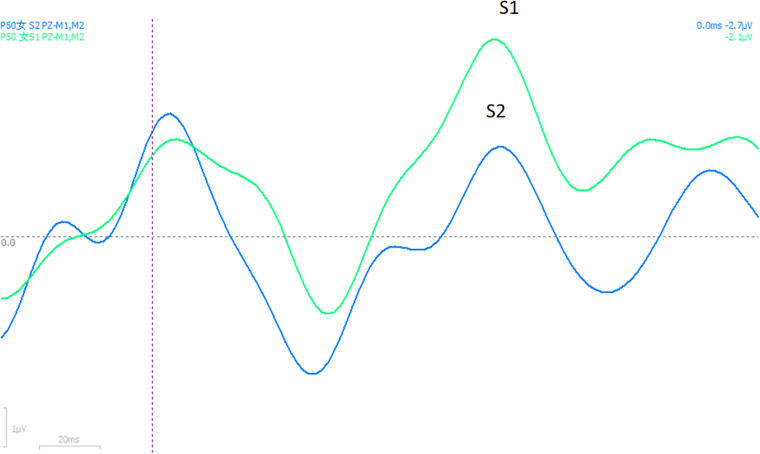
Suppression ratio of amplitude (S2:S1) for P50.

Although there is no difference in gender between the insomnia group and control group, significant gender difference in ASSR latency has been found in the insomnia group located in T7 (Figure 1) and PZ (Figure 2), no matter whether 20 or 40 Hz ASSR is used (T7 represents as middle temporal region). 20 Hz ASSR latency located in T7: male 329.20 ± 22.09 vs. female 351.00 ± 30.02, *P* = 0.048; 40 Hz ASSR latency located in T7: male 322.40 ± 19.84 vs. female 342.91 ± 28.47, *P* = 0.048; 20 Hz ASSR latency located in PZ: male 350.09 ± 33.52 vs. female 331.52 ± 19.66, *P* = 0.05; 40 Hz ASSR latency located in PZ: male 362.36 ± 36.56 vs. female 339.68 ± 22.85, *P* = 0.036). Additionally, suppression ratio of amplitudes (S2:S1) for P50 varied between genders, especially located in Pz (male 1.75 vs. female 0.59, *P* = 0.044). For the suppression ratio of amplitude (S2:S1) for P50 see Figure 3.

### Evaluation Between Sleep Characteristics and ASSR, P50 in Insomnia Group

**FIGURE 4:**
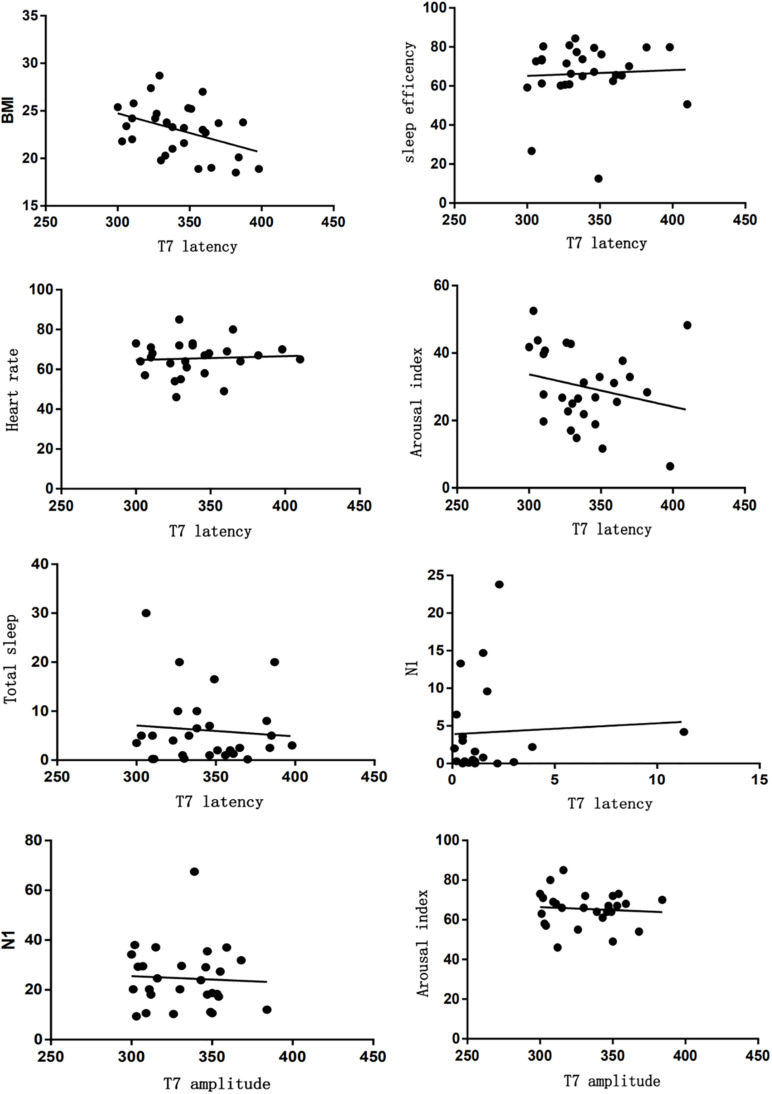
Correlation analysis between sleep characteristics and ASSR located in T7.

**FIGURE 5:**
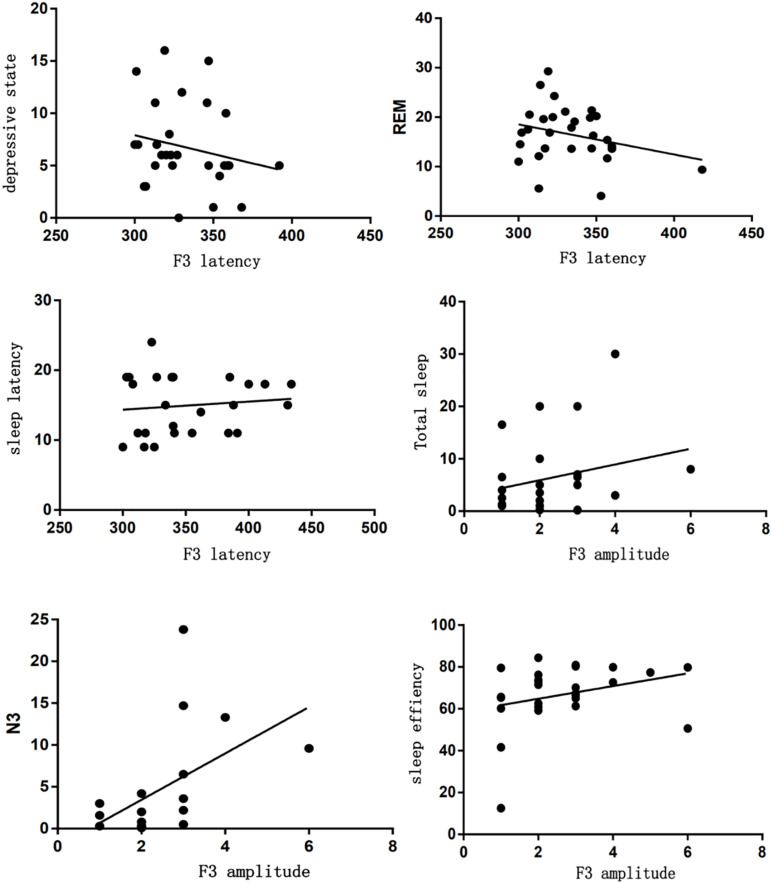
Correlation analysis between sleep characteristics and ASSR located in F3.

As shown, there was a significant difference between sleep characteristics and ASSR parameters. 20 Hz ASSR latency located in T7 was correlated with depressive tendency (*r* = −0.474), BMI (*r* = −0.439), sleep efficiency (*r* = −0.35), arousal index (*r* = −0.459), TST (*r* = −0.399), N1 (*r* = −0.458), and heart rate (*r* = −0.389). 40 Hz ASSR amplitude located in T7 was correlated with depressive tendency (*r* = 0.315), arousal index (*r* = 0.399), N1 (*r* = 0.513), and heart rate (*r* = −0.36) (Figure 4). 40 Hz ASSR latency or amplitude located in F3 has correlation analysis; for example, 40 Hz ASSR latency was correlated with depressive tendency (*r* = 0.364), sleep latency (*r* = −0.388), and REM (*r* = −0.346). 40 Hz ASSR amplitude was correlated with sleep efficiency (*r* = −0.491), TST (*r* = −0.366), and N3 (*r* = −0.44) (Figure 5).

**FIGURE 6:**
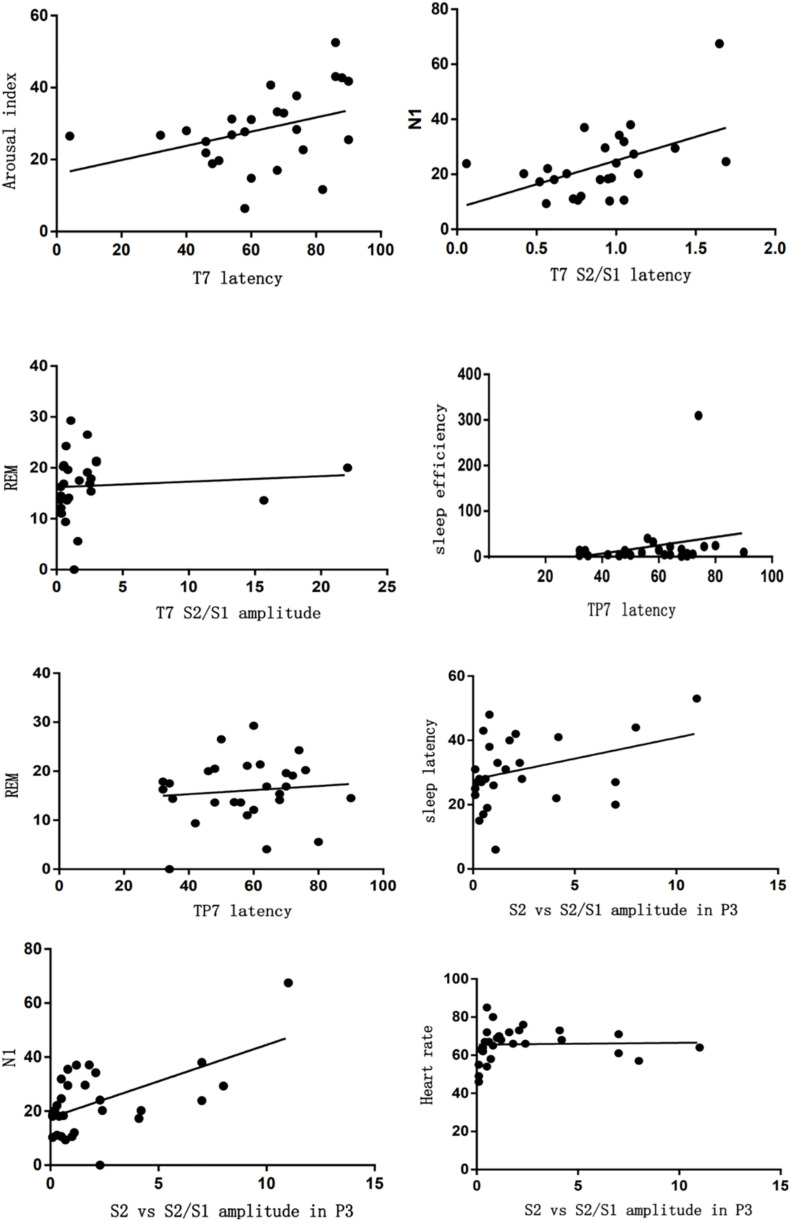
Correlation analysis between sleep characteristics and P50 located in left temporal parietal lobe.

**FIGURE 7:**
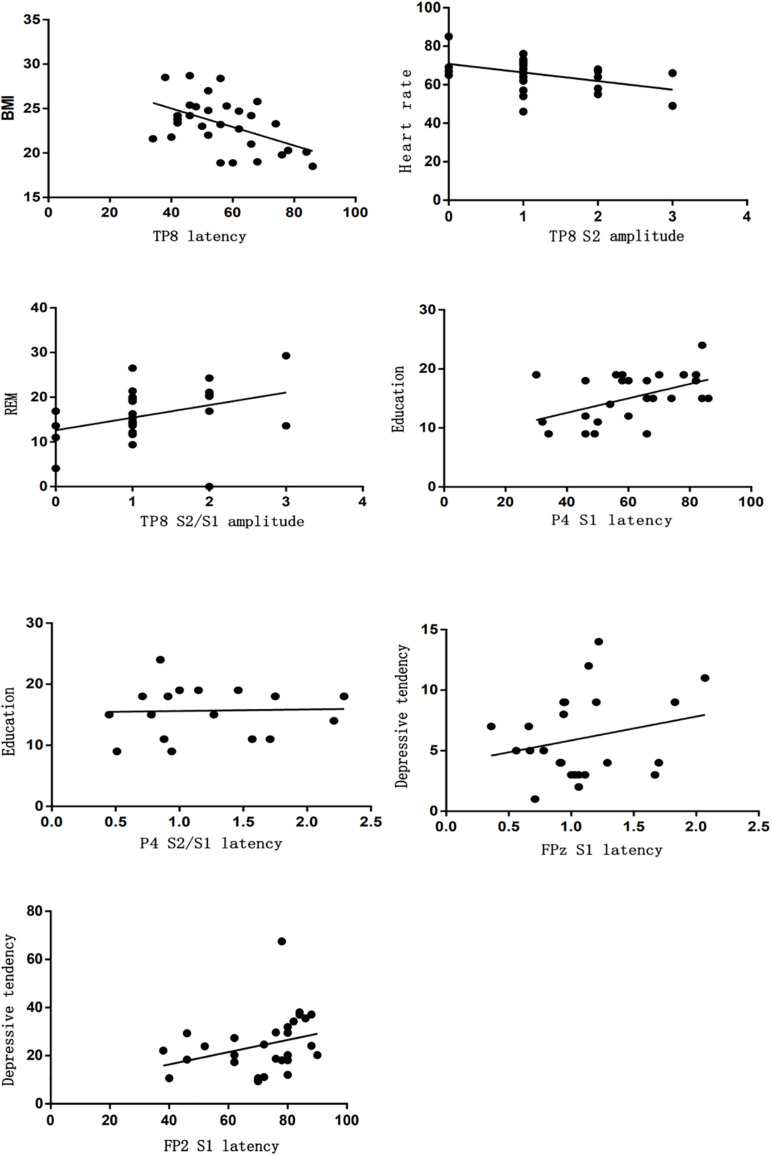
Correlation analysis between sleep characteristics and P50 located in right temporal parietal lobe.

Additionally, the latency or amplitude of P50 located in left temporal parietal lobe (T7, TP7, and P3) was correlated with sleep characteristics, such as the latency of S2/S1 or S1 located in T7 was correlated with arousal index (*r* = 0.418, *r* = 0.371). The latency of S2/S1 located in T7 was correlated with N1 (*r* = 0.407). The amplitude of S2/S1 located in T7 was correlated with REM (*r* = 0.419). The latency of S1 located in TP7 was correlated with sleep latency and REM (*r* = 0.392, *r* = 0.372). The amplitude of S2 vs. S2/S1 located in P3 was correlated with arousal index, N1, and heart rate (*r* = −0.383 vs. −0.425, −0.548 vs. −0.551, −0.352 vs. −0.481) (Figure 6). The latency or amplitude of P50 located in right temporal parietal lobe (TP8, P4) was correlated with sleep characteristics, such as the latency of S1 located in TP8 was correlated with BMI (*r* = −0.482) and the amplitude of S1or S2/S1 located in TP8 was correlated with heart rate and REM, respectively (*r* = −0.37, *r* = −0.479). The latency of S1 or S2/S1 located in P4 was correlated with education (*r* = 0.543, 0.449). In addition, latency of P50 located in frontal lobe (FPz, FP2, and F4) was correlated with depressive tendency, especially with the S1 latency located in FPz, FP2 (*r* = 0.346, *r* = 0.55) and S2/S1 latency located in FP2, F4 (*r* = 0.341, *r* = 0343) (Figure 7).

### Evaluation of MMN Between Insomnia Group and Control Group

**FIGURE 8:**
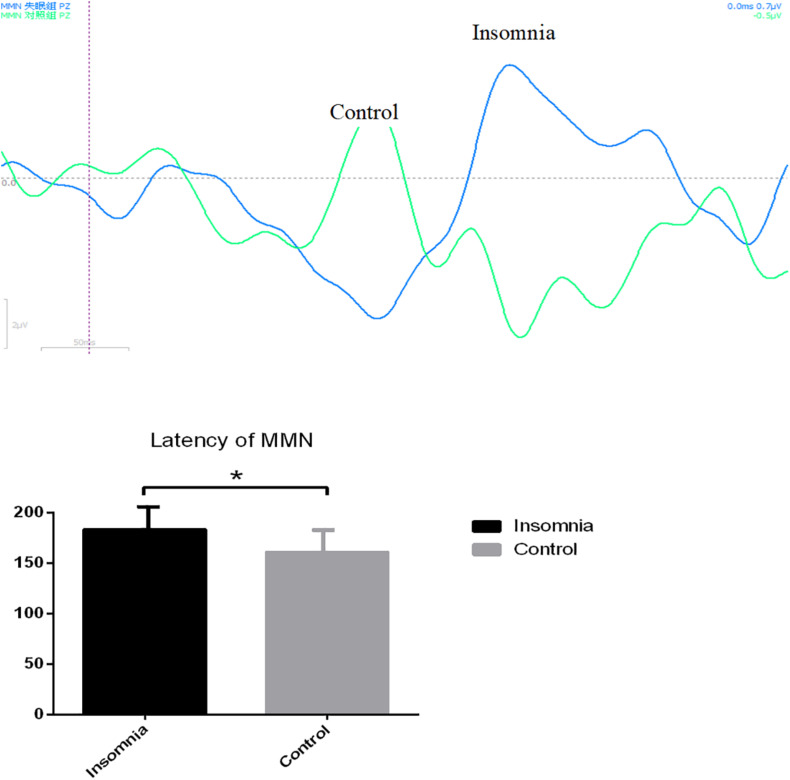
The latency of mismatch negativity (MMN) between insomnia group and control group. ^∗^*P* < 0.05.

There was a significant difference of MMN latency between the insomnia group and control group; the latency of MMN in the insomnia group was much longer than in the control group (183.65 ± 40.200 vs. 161.43 ± 40.882, *P* = 0.049) (Figure 8). Additionally, there was a higher tendency of depression found in the insomnia group vs. control group (*P* = 0.008). And MMN latency located in midline areas such as F_*Z*_, C_*Z*_, and P_*Z*_ was significantly correlated with depressive tendency (*P* = 0.008, *P* = 0.003, and *P* = 0.006).

### Evaluation Between Gender and MMN in Insomnia Group

Although there is no difference in gender between the insomnia group and control group, significant gender difference in MMN amplitude has been found in the insomnia group located in FPz, Cz, and Pz (MMN amplitude located in FPz: male 1.36 ± 0.96 vs. female 2.58 ± 1.76, *P* = 0.038; MMN amplitude located in Cz: male 131.00 ± 29.56 vs. female 162.44 ± 42.47, *P* = 0.033; MMN amplitude located in Pz: male 1.45 ± 1.50 vs. female 2.86 ± 2.00, *P* = 0.045).

### Evaluation Between Sleep Characteristics and MMN in Insomnia Group

As shown, there was a significant difference between sleep characteristics and MMN parameters located in midline areas. The amplitude of MMN located in Fz was correlated with BMI, sleep efficiency, and N1 (*r* = 0.038, *r* = 0.012, and *r* = 0.019). The amplitude of MMN located in Cz was correlated with BMI, N1, and N3 (*r* = 0.038, *r* = 0.012, and *r* = 0.019). The amplitude of MMN located in Pz was correlated with BMI, sleep efficiency, and N3 (*r* = 0.031, *r* = 0.036, and *r* = 0.007). In addition to this, the MMN latency located in right temporal lobe as T8 and TP8 was correlated with N1 and N2. The MMN latency located in T8 was correlated with N1 and N2 (*r* = 0.032, *r* = 0.01), and the MMN latency located in Tp8 was correlated with N1 and N2 (*r* = 0.038, *r* = 0.07).

## Discussion

The major finding of this study is that gender influenced AASR latency in insomnia. No matter whether 20 or 40 Hz ASSR was used, there was a significant difference found between females and males. Aside from interference factors such as the samples and the methods applied (in our study methods were consistent with previous studies), gender has been shown to influence a variety of electrophysiological results. In previous studies, it has been widely reported that women have larger brainstem and cortical evoked responses (Fein and Brown, 1987; Shearer et al., 1992) and shorter evoked potential (EP) latency than men (Trune et al., 1988; Taylor et al., 1990). Although, the relevance of the observed gender differences to pathophysiology is unclear, but anatomical and cortical organizational differences between the genders may contribute to the observed differences. The effect of menstruation, reproductive hormones, and steroids to EP has been extensively investigated (Waldo et al., 1987). Additionally, the difference between the genders may be affected by differential arousal levels observed in the men and women, such as, women may be less tolerant to loud tones than men and be more distractable when presented with aural stimuli (McGuinness, 1974; Halley, 1975). Although, there are scattered studies of the effects of gender on auditory EP, such as recovery cycle, distractibility, or EP augmenting-reducing, but no difference between the genders was found for visual stimuli (Cohn et al., 1985).

Except for gender difference, reduced ASSR (particularly 40 Hz ASSR) may reflect a loss of neural integration and may be an indicator of cognitive deficits, which was related with patients with psychiatric disorders (Spencer et al., 2008, 2009). As shown in our study, 40 Hz ASSR located in T7 or F3 was correlated with depressive tendency, which was consistent with Hall’s study (Zhou et al., 2018). Additionally, 40 Hz ASSR located in T7 or F3 was negatively correlated with sleep parameters, such as sleep efficiency or arousal index. Such results obtained may be explained by the physiological basis of ASSR. AASR generated in primary auditory cortex and was widely used to assess neural oscillations, especially at gamma band frequencies (30–100 Hz). Neural oscillations were thought to play an important role in signal transmission and integration of information across cortical networks (Uhlhaas et al., 2008). Such deficits of neural oscillations were reported to stem from the GABAergic dysfunction. And GABAergic neural function was associated with normal sleep and emotional stability. Thus, ASSR deficits can be used as a representative tool to detect sleep disorders or emotional disorders (Gonzalez-Burgos and Lewis, 2008). ASSR deficits were also selective, and not significant at lower stimulation frequencies such as 20 Hz.

According to P50 results, a relationship was found between P50 gating and education, such as increased intellectual capability and stronger P50 gating, which may be interpreted as a protective function of the integrity of higher-order functions (Boutros et al., 2004; Wan et al., 2008). Sensory gating refers to the ability of cerebral networks to transmit the incoming information and filter out irrelevant stimuli, which protects the human brain from information overflow. Thus, there is a close association between P50 and education. However, such a result needs further investigation.

Sensory gating P50 is an electrophysiological index which reflects the brain inhibiting function. Normal cerebrum has a function of selecting and filtering stimulus, and admits significant information into the more senior centrum by sensory gating. In this way, sensory overload can be avoided, but a defect of sensory gating will induce the overload of indifferent stimulus and all kinds of psychiatric symptoms correlated with attention (Wang et al., 2009). In a previous study Sussman et al. (2014) reported that patients with depression also had deficits of P50 suppression and they could not filter irrelevant information effectively. As shown in our study, the latency of P50 (S1 latency or S2/S1 latency) located in the frontal lobe was significantly correlated with depressive tendency, which indicated that insomnia patients with depressive tendencies had defects in information processing and focused on indifferent stimulations in their surroundings. In clinical work, we also found that such insomnia patients showed an insufficient reaction to something which was important to them. We supposed that they should have deficits on P50.

Additionally, P50 has an association with sleep parameters, such as arousal index, N1, and REM. However, the latency of P50 has a positive correlation with such sleep parameters, while the amplitude of P50 has a negative correlation with them. It may be explained by the fact that bad sleeping with higher arousal and shorter N1or REM will cause longer P50 latency and lower amplitude. Such results may indicate that insomnia patients with poor sleeping will disturb cognitive function.

Mismatch negativity is an involuntary brain response to auditory change or, more generally, to regularity violation. MMN enables one to explore the neurobiological substrate of central auditory processing, particularly into auditory memory (Sussman et al., 2014). Previous studies have reported that attenuated MMN amplitudes to auditory contrasts have been found in mild cognitive impairment (Mowszowski et al., 2012). Some studies have suggested that the greater the MMN deficit, the weaker the cognitive or functional status of the patients (Kaur et al., 2013). Such as in our study, the latency of MMN in insomnia was much longer than controls, which may indicate weaker cognitive function in insomnia patients. In addition, MMN latency located in midline areas was significantly correlated with depressive tendency, also shown in other studies’ results (He et al., 2010). Such results may be explained as a condition of decreased cortical neuronal excitability in depressive tendency, due to the deficit of GABAergic dysfunction.

Furthermore, the amplitude of MMN located in the midline areas, especially located in anterior head, was significantly correlated with sleep parameters, such as sleep efficiency, N1, and N3. And the amplitude of MMN located in the right temporal lobe was correlated with N1 and N2. As the results show, anterior areas and the right temporal lobe may be important local regions to the relationship between sleep quality and cognitive function. To explain this finding, it has been suggested that both frontal MMN generators and temporal generators are significant in the regulation of MMN activity. In this light, the main role of frontal MMN generators is a top-down control on the mismatch detection mechanism, when the activity of these frontal networks is impaired, the activity of the temporal generators is deregulated (Restuccia et al., 2016). Thus, poor sleep may affect cognitive function in insomnia patients and manifest as dysfunction of MMN activity located in anterior and temporal locations.

Above all, in our study, several limitations need to be taken into account. Firstly, the small number of subjects enrolled may make it impossible to establish a reliable correlation between clinical and neurophysiological findings. Secondly, it is worth enrolling such patients as a subtype or subgroup and it is also worth trying such ERP tasks during the night, not only during the day. Thirdly, insomnia patients only have one night in the monitoring room with one night PSG analysis. Under this condition, the results may be affected by the first night effect. Nevertheless, a multiple night design may be considered in future studies. Finally, it may not be sufficient to exclude the potential effect of apnea-hypopnea or periodic leg movement event on the FFT analysis. In our exclusion criteria, we excluded those patients or controls with an AHI >5 and PLMS >15. Therefore, in future studies, we may improve electrophysiological techniques to exclude the artifacts and eliminate the negative effects.

## Conclusion

Our results suggested robust electrophysiological abnormalities, such as ASSR, P50, and MMN, in insomnia patients. Such abnormalities included gender difference, education difference, difference in depressive tendency, and difference in sleep parameters. The results revealed varied cognitive dysfunction involving inputs and processing in insomnia patients. And at the same time, we also explored the neuropsychological mechanisms underlying the cognitive dysfunction with different ERP tasks.

## Data Availability Statement

The raw data supporting the conclusions of this article will be made available by the authors, without undue reservation.

## Ethics Statement

The studies involving human participants were reviewed and approved by the Ethics committee of Jilin University First Hospital. The patients/participants provided their written informed consent to participate in this study.

## Author Contributions

CL and HZ were involved in the acquisition of data and analysis and interpretation of data. YL made substantial contributions to the conception and design, drafting the manuscript, and revising it critically for important intellectual content. All authors have given their final approval of the version to be published. Each author has participated sufficiently in the work to take public responsibility for appropriate portions of the content and agreed to be accountable for all aspects of the work in ensuring that questions related to the accuracy or integrity of any part of the work are appropriately investigated and resolved.

## Conflict of Interest

The authors declare that the research was conducted in the absence of any commercial or financial relationships that could be construed as a potential conflict of interest. The reviewer YZ declared a shared affiliation, with no collaboration, with several of the authors YL, HZ, CL, and XZ to the handling editor at the time of the review.

## Publisher’s Note

All claims expressed in this article are solely those of the authors and do not necessarily represent those of their affiliated organizations, or those of the publisher, the editors and the reviewers. Any product that may be evaluated in this article, or claim that may be made by its manufacturer, is not guaranteed or endorsed by the publisher.
